# Relevance of the Induced Stress Resistance When Identifying the Critical Microorganism for Microbial Risk Assessment

**DOI:** 10.3389/fmicb.2018.01663

**Published:** 2018-07-24

**Authors:** Alberto Garre, Jose A. Egea, Asunción Iguaz, Alfredo Palop, Pablo S. Fernandez

**Affiliations:** ^1^Departamento de Ingeniería de Alimentos y del Equipamiento Agrícola, Instituto de Biotecnología Vegetal, Universidad Politécnica de Cartagena (ETSIA), Cartagena, Spain; ^2^Departamento de Matemática Aplicada y Estadística, Universidad Politécnica de Cartagena, Antiguo Hospital de Marina (ETSII), Cartagena, Spain

**Keywords:** microbial inactivation, induced stress resistance, bacterial acclimation, *E. coli*, thermal inactivation

## Abstract

Decisions regarding microbial risk assessment usually have to be carried out with incomplete information. This is due to the large number of possible scenarios and the lack of specific data for the problem considered. Consequently, risk assessment studies are based on the information obtained with a small number of bacterial cells which are considered the most heat resistant and/or more capable of multiplying during storage. The identification of the most resistant strains is usually based on D and z-values, normally estimated from isothermal experiments. This procedure omits the potential effect that the shape of the dynamic thermal profile applied in industry has on the microbial inactivation. One example of such effects is stress acclimation, which is related to a physiological response of the cells during sub-lethal treatments that increases their resistance. In this article, we use a recently published mathematical model to compare the development of thermal resistance for *Escherichia coli* K12 MG1655 and *E. coli* CECT 515 using inactivation data already published for these strains. Based only on the isothermal experiments, *E. coli* K12 MG1655 would be identified as more resistant to the thermal treatment than the CECT 515 strain in the 50–65°C temperature range. However, we conclude that stress acclimation is strain (and/or media)-dependent; the CECT 515 strain has a higher capacity for developing a stress acclimation than K12 MG1655 (300% increase of the D-value for CECT 515, 50% for K12 MG1655). It, thus, has the potential to be more resistant to the thermal treatment than the K12 MG1655 strain for some conditions allowing acclimation. A methodology is proposed to identify for which conditions this may be the case. After calibrating the model parameters representing acclimation using real experimental data, the applicability of the proposed approach is demonstrated using numerical simulations, showing how the CECT 515 strain can be more resistant for some heating profiles. Consequently, the most resistant bacterial strain to a dynamic heating profile should not be identified based only on isothermal experiments (D- and z-value). The relevance of stress acclimation for the treatment studied should also be evaluated.

## Introduction

Microbiological risk assessment tries to estimate the probability that the consumer of a food product contracts a sickness due to the presence of a pathogen in the product above a critical concentration (Allende et al., 2018). This is a complex task, because of the high relevance of uncertainty and variability, as well as the need to deal with incomplete data (Zwietering, 2009). The last years have witnessed a leap forward in the methodologies used for microbiological risk assessment, going from qualitative to quantitative methods (Coleman and Marks, 1999). Quantitative methodologies are based on a quantitative description of the microbial response to the conditions that the food product may encounter during its life cycle (e.g., storage and processing) and usually provide a more accurate estimate of the risk of exposure than qualitative methods (Koutsoumanis et al., 2016). These quantitative methodologies are usually based on predictive microbiology, which applies mathematical modeling to describe the microbial response during conditions that may allow its grow or result in microbial inactivation (Perez-Rodriguez and Valero, 2012).

Nevertheless, mathematical models used in predictive microbiology have a strong empirical nature, having some model parameters that must be estimated based on experimental data before its use for prediction. Several studies have pointed out the variability in the bacterial response of different strains when exposed to similar conditions, expressed by different values of the model parameters characterizing the microbial response (Nauta, 2000; Hassani et al., 2006; van Asselt and Zwietering, 2006; Bruschi et al., 2017). This is an issue for microbial risk assessment, due to the large number of different microbial strains that can potentially contaminate a good product. As an illustrative example, ComBase (Baranyi and Tamplin, 2004) includes inactivation and/or growth parameters for over 40 bacteria taxa, which can be further subdivided (e.g., *Escherichia coli* has 180 different serogroups Stenutz et al., 2006). Therefore, risk assessment cannot be performed for every potential strain using the resources available nowadays. Instead, it is limited to some bacterial strains identified as the most resistant ones and/or the ones with the highest growth potential.

The most resistant bacterial strains are usually identified based on the model parameters describing their response to the processing conditions: the D and z-values. The D-value is the time required to apply a constant thermal stress to inactivate a 90% of the bacterial population of a particular strain, whereas the z-value is the temperature increase needed to cause a 10-fold reduction of the D-value. These parameters cannot be known beforehand and must be experimentally estimated (or are taken from databases based on experimental data). Several review articles (van Asselt and Zwietering, 2006; Cebrián et al., 2016; Petruzzi et al., 2017) gather the model parameters of the most relevant microorganism(s) for food safety in different conditions. Nonetheless, the characterization of microbial inactivation is still an active field of research due to the emergence of new processing technologies (Knorr, 2018) and food products (Aertsens et al., 2009; O'Shea et al., 2012; Bigliardi and Galati, 2013; Liu et al., 2015; González-Tejedor et al., 2017; Klug et al., 2018). Once the D and z-values are identified, the efficacy of a thermal treatment is evaluated using the cumulative F-value (Stumbo, 1973). Nevertheless, during the last years several authors have criticized this approach because it does not takes into account non-linearities in the microbial response such as shoulder and tail effects (Peleg, 2006).

The study of the microbial response has been commonly undertaken using isothermal experiments. These results have, then, been extrapolated to describe non-isothermal processes applied in industrial conditions (such as retorts or heat exchangers). However, several articles have highlighted the problems associated to this approach (Hassani et al., 2005; Valdramidis et al., 2006; Janssen et al., 2008; Stasiewicz et al., 2008). A particular case is stress acclimation (also referred to as stress adaptation or induced stress resistance). Thermal treatments, such as those used for pasteurization, begin at sub-lethal temperatures. If the heating rate of the food substrate is not fast enough, physiological changes may occur in the bacterial cells, increasing their resistance to posterior stresses (Hill et al., 2002; Richter et al., 2010). As a consequence, a higher number of bacterial cells will be able to survive the treatment with respect to the one predicted based on isothermal experiments, even several log-units higher than expected (Valdramidis et al., 2006; Hassani et al., 2007). It is, thus, a potential food safety risk because a larger number of pathogenic bacterial cells than expected may survive the treatment.

Several models were proposed during the last 10 years to describe the kinetics of stress acclimation (Dolan and Mishra, 2013). Recently, Garre et al. (2018) proposed a novel mathematical model, based on the Bigelow log-linear model, to describe this phenomenon. One of the advantages of this model with respect to the previous ones is that it makes an explicit difference between “static” thermal resistance (the one due to environmental conditions at each time point) and “dynamic” resistance (stress acclimation). It does so by including a variable describing a hypothetical physiological state of the microbial cells, which quantifies the level of stress developed by the them. Hence, it provides further insight on how the stress acclimation could be developed during a non-isothermal treatment. In this article, we illustrate how the formulation of this model allows a comparison of the ability of two different strains of *E. coli* to increase their resistance to thermal stresses. Furthermore, we explore how stress acclimation may be relevant for microbial risk assessment, studying how it can influence the choice of the most resistant bacterial strain to a particular thermal treatment.

## Materials and methods

### Microbiological data

**Figure 1:**
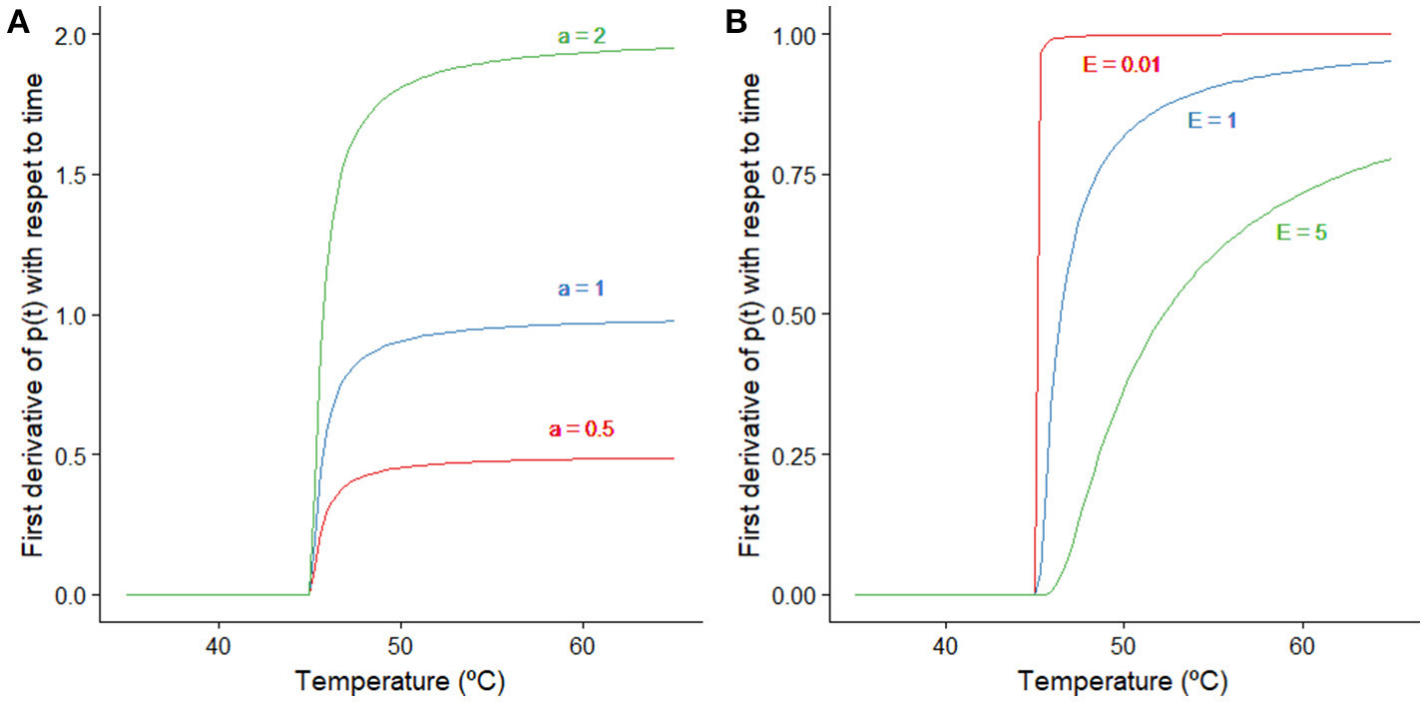
Representation of the effect that changes on the model parameters *a* and *E* have on Equation (4). **(A)** Effect of the parameter *a* when the remaining parameters are kept constant (*p(t)* = *0, E* = *0.5*°*C, T*_*si*_ = *45*°*C*). **(B)** Effect of the parameter *E* when the remaining parameters are kept constant (*p(t)* = *0, a* = *1 min*^−1^, *T*_*si*_ = *45*°*C*).

The data on microbial inactivation of *E. coli* K12 MG1655 reported by Valdramidis et al. (2006) and of *E. coli* CECT 515 reported by Garre et al. (2018) were used in this study. Valdramidis et al. (2006) characterized the isothermal and non-isothermal bacterial inactivation experiments using Brain Heart Infusion (BHI) broth as heating medium. The isothermal experiments were performed using capillary tubes immersed in a circulating water bath (GR150-S12, Grant) at 49.5, 52, 54, 54.6, 55, 56.6, 58.6, and 60.6°C. The non-isothermal experiments were performed in a similar fashion. Using the Labwise© program, six different dynamic profiles were programmed. All of them were biphasic with an initial heating phase with varying intensity (0.15, 0.20, 0.40, 0.55, 0.82, and 1.64°C/min) and a holding phase at 55°C (see solid line in Figure 1). For further details on the experimental procedure please refer to the original article by Valdramidis et al. (2006). The experimental results were made available to us by the authors as text files.

Garre et al. (2018) used *E. coli* CECT 515 supplied by the Spanish Type Culture Collection. These authors performed isothermal and non-isothermal experiments using Peptone water (PW) as heating medium in a Mastia thermoresistometer (Conesa et al., 2009). Isothermal experiments were carried out at 52.5, 55, 57.5, and 60°C, whereas two families of non-isothermal profiles were tested: monophasic profiles with a constant heating, and biphasic profiles similar to the ones tested by Valdramidis et al. (2006). Different heating rates were used, ranging from 1 to 40°C/min. Garre et al. (2018) analyzed their data using the same model that has been used in this study. Hence, the model parameters reported by them have been reused here.

### Mathematical modeling of microbial inactivation

The inactivation of *E. coli* during the non-isothermal treatments was described using the mathematical model proposed by Garre et al. (2018). This model is based on the classical first order kinetics model Equation (1**)**, which considers that the thermal resistance of the microbial cells is homogeneous within the population. As a consequence, the time that single cells can resist a constant stress are independent identically distributed random variables and the first derivative of the microbial density (*N*) at time *t* is proportional to itself. The proportionality constant is given by the inactivation rate (*k(T)*).

(1) $$\begin{array}{r} \frac{dN}{dt}=-k\left(T\right)\cdot N \end{array}$$

In predictive microbiology, the D-value (*D*(*T*) = ln 10/*k*(*T*)) is commonly used instead of the inactivation rate, k. This parameter defines the time that is required to keep a constant stress to reduce the microbial density a 90%. The D-value is usually considered to have an exponential relationship with temperature (Bigelow, 1921), as shown in Equation (2). The parameter *z* (usually called z-value) describes the temperature increment required to reduce the D-value a 90%. This model makes use of a reference temperature (*T*_*ref*_) without any biological meaning, but with an impact on model identifiability (Dolan and Mishra, 2013). Note how the relationship between the D-value and temperature does not take into consideration the heating history of the microbial population and, thus, is not able to describe the induced stress resistance.

(2) $$\begin{array}{r} D\left(T\right)=D\left(T_{ref}\right)\cdot 10^{-\frac{T-T_{ref}}{z}} \end{array}$$

The model proposed by Garre et al. (2018) modifies the inactivation rate defined in Equation (1) in order to reflect the heating history. It considers that the inactivation rate (a function of both time and temperature) equals the product of two terms, as shown in Equation (3). The first one (*k*_1_) describes the thermal resistance due to the environmental conditions at each time point, i.e. without any consideration for the heating history of the bacterial cells. In this model, this term is equivalent to the one defined by Bigelow. The heating history of the bacterial cells, which results in an acclimation, is modeled by the second term (*k*_2_), whose algebraic form is defined in Equation (3). It uses one variable, *p(t)*, and one model parameter (*c*). The variable *p(t)* describes a hypothetical physiological state of the cells with respect to the acclimation. When *p*(*t*) = 0 bacterial cells have not developed any acclimation. In that case, *k*_2_(*t*) = 1 and the model predicts the same inactivation rate that would be predicted by the Bigelow model (i.e., the value of *k*_1_(*T*)). In the model by Garre et al. (2018), the variable *p(t)* has an upper bound of one. The case when *p*(*t*) = 1 assumes that the cell has developed its maximum acclimation. In this case, the inactivation rate predicted by the model is *k*(*t, T*) = *k*_1_/(1 + *c*). In other words, the D-value predicted by the Bigelow model is increased by a factor equal to (1 + *c*). Consequently, the model parameter *c* describes the effect that the bacterial acclimation has on the inactivation rate. For instance, if *c* = 1, the bacterial acclimation can potentially increase the D-value predicted by *k*_1_(*T*) by a factor of two; *i.e*. it can double the D-value. Note that *p(t)* is a continuous variable. Therefore, the bacterial acclimation in the model by Garre et al. (2018) is not a discrete phenomenon, and therefore it can model intermediate levels of adaptation when *p(t)* takes values between zero and one. According to this, a value of *p*(*t*) = 0.5 would mean that the bacteria has reached a 50% of its maximum capacity to develop an stress acclimation.

(3) $$\begin{array}{rcl} \frac{dN}{dt} & = & k\left(t,T\right)N\left(t\right)=k_{1}\left(T\right)\cdot k_{2}\left(t\right)N\left(t\right)\\ = & \frac{\ln\;10}{D\left(T_{ref}\right)\cdot 10^{-\frac{T-T_{ref}}{z}}}\cdot \frac{1}{1+c\cdot p\left(t\right)}N\left(t\right) \end{array}$$

This model considers that variable *p(t)* describing the adaptation only increases its value when the bacterial cells are stressed. That is, when the temperature of the treatment is above a stress inducing temperature (*T*_*si*_). If this is the case, this variable increases exponentially with temperature until *p*(*t*) = 1 when its value remains constant, as shown in Equation (4). The magnitude of the first derivative of *p(t)* with respect to time depends on the values of the model parameters *a* and *E*. Figure 1 illustrates how changes in those parameters affect the inactivation rate when the remaining parameters are kept constant. For temperatures much higher than *T*_*si*_, the exponential term tends to one and $\frac{dp}{dt}$ equals the model parameter *a* (for low *p(t)* values). The model parameter *E* quantifies how fast the transition between both regimes; lower values of *E* imply a faster transition.

(4) $$\frac{dp}{dt}=\{\begin{array}{rr} 0, & T<T_{si}\\ a\;\cdot \;e^{-\frac{E}{T-T_{si}}}(1-p(t)), & T\geq T_{si} \end{array}$$

### Model fitting of non-isothermal experiments and numerical predictions

The model fitting was performed following the two-step procedure suggested by Garre et al. (2018). In a first step, the D- and z-values are estimated using data generated under isothermal experiments. Next, the model parameters describing the development of a stress acclimation (*a, c* and *E*) are estimated using one non-isothermal experiment, except the stress inducing temperature (*T*_*si*_) which was fixed to the maximum temperature for growth of *E. coli* (45°C). This procedure has several advantages with respect to a procedure where the five model parameters are fitted in one step. Firstly, potential structural identifiability issues due to parameter correlations are avoided (Vilas et al., 2018). Secondly, this allows to reuse D and z-values that have already been published in the literature based on isothermal experiments, reducing the experimental effort required to fit the model. Consequently, the D and z-values were not estimated. Instead, the ones reported by Valdramidis et al. (2006) for isothermal experiments using the same strain and heating media than the ones used for dynamic experiment (*D*_56.3_ = 5.67*min* (*sd* = 0.61), *z* = 4.11*oC* (*sd* = 0.16)) were used. The three remaining parameters were estimated from the experimental data obtained from a single dynamic experiment using the Adaptive Monte Carlo algorithm by Haario et al. (2006), implemented in the FME package (Soetaert and Petzoldt, 2010). The remaining non-isothermal profiles were set aside for model validation. The convergence of the fitted parameters was evaluated following the guidelines by Brooks et al. (2011). A trace and running mean plot was used to evaluate the quality of the mixing and the stability of the solution, whereas the lack of correlation was checked using an autocorrelation plot. Moreover, the test by Heidelberger and Welch (1983) was used to ensure the stationarity of the solution. A total of 8000 iterations of the MCMC chain, with a burninglength of 1000 iterations, were required to achieve convergence. Garre et al. (2018) already fitted this mathematical model to their data, so the model parameters reported in this study were used.

The non-isothermal microbial inactivation predicted by the Bigelow model based on isothermal experiments was calculated using the *bioinactivation* package for R (Garre et al., 2017). For the acclimation model by Garre et al. (2018), the differential equation was solved using *lsoda* algorithm (Hindmarsh, 1983), implemented in *deSolve* package for R (Soetaert et al., 2010).

The goodness of fit was evaluated using the Mean Error (*ME*) and Root Mean Squared Error (*RMSE*) commonly used in statistics literature. The *ME* Equation (5) is the mean difference between the model predictions ($\log_{10}\hat{N}$) and the *n* experimental observations (log_10_*N*). Hence, values of *ME* greater than zero indicate that the model systematically underpredicts the response (i.e., overpredicts microbial inactivation), with greater values of the *ME* indicating a larger bias. Values smaller than zero indicate the opposite (i.e., underpredicts the microbial inactivation), whereas an *ME* equal to zero indicates the absence of bias. The *RMSE* (Equation (6)) indicates the dispersion of experimental observations with respect to model predictions. When the RMSE equals zero, the model predicts the experimental observations without any error, whereas higher values imply a lower precision.

(5) $$\begin{array}{rcl} ME & = & \frac{1}{n}\underset{i}{\sum }\left(\log_{10}\hat{N_{i}}-\log_{10}N_{i}\right) \end{array}$$

(6) $$\begin{array}{rcl} RMSE & = & \sqrt{\frac{1}{n}\underset{i}{\sum }{\left(\log_{10}\hat{N_{i}}-\log_{10}N_{i}\right)}^{2}} \end{array}$$

Moreover, the Accuracy factor (*A*_*f*_) and Bias factor (*B*_*f*_), commonly used in predictive microbiology were calculated. These indexes were first defined by Ross (1996) and later refined by Baranyi et al. (1999). The Accuracy factor Equation (7) has a similar interpretation as the *RMSE*, indicating the precision of the model predictions. However, it is defined between one and plus infinite, with values equal to one being identified with a perfect fit, whereas higher values indicate lower precision. The Bias factor Equation (8) is analogous to the *ME*, assessing the bias of the model predictions. Values of this index between zero and one indicate that the model systematically underpredicts the microbial density, whereas values between one and plus infinite mean the opposite. The absence of bias results in a bias factor equal to one.

(7) $$\begin{array}{rcl} A_{f} & = & 10^{\sqrt{\frac{1}{n}\sum_{i}{\left(\log_{10}\hat{N_{i}}-\log_{10}N_{i}\right)}^{2}}} \end{array}$$

(8) $$\begin{array}{rcl} B_{f} & = & 10^{\frac{1}{n}\sum_{i}\left(\log_{10}\hat{N_{i}}-\log_{10}N_{i}\right)} \end{array}$$

### Generation of induced thermal resistance diagram

Garre et al. (2018) suggested the use of a two-dimensional diagram to visualize how the development of a stress tolerance varies with the thermal inactivation profile. This diagram represents the D-value (*D*_*h*_) predicted (considering the adaptation) after a heating phase with heating rate (*HR*) constant, until a temperature (*T*) is reached. This is calculated as shown in Equation (9), where *p*_*h*_ is the value of *p(t)* predicted by the model at the end of the heating phase. The repetition of this calculation for different values of *T* and *HR* results in three-dimensional surface which describes how the stress acclimation is developed. This surface can be visualized in a 2-dimensional plane, joining with solid lines combinations of both factors (*HR* and *T*) resulting in the same D-value, allowing the visualization of how the ability of the bacterial cells to develop a stress resistance is affected by the duration of the heating phase and the maximum temperature.

(9) $$\begin{array}{r} D_{h}=\left(D\left(T_{ref}\right)\cdot 10^{-\frac{T-T_{ref}}{z}}\right)\left(1+c\cdot p_{h}\right) \end{array}$$

## Results and discussion

Data from Valdramidis et al. (2006) and from our group were analyzed using a recently published model (Garre et al., 2018) in order to estimate the adaptation capacity of two strains of *E. coli* when exposed to non-isothermal heat treatments that included sublethal temperatures. To fit the model, a D-value of 5.67 min (sd = 0.61) at 56.3°C and a z-value of 4.11°C (sd = 0.16) for *E. coli* K12 MG1655 on isothermal experiments using BHI as heating medium were considered as reported by Valdramidis et al. (2006). Garre et al. (2018) propose to fit the parameters *D*(*T*_*ref*_), *T*_*ref*_ and *z* of their model Equation (3) to those estimated using isothermal experiments because under isothermal conditions bacterial cells are inoculated at the target temperature. Therefore, inactivation takes place before they can develop an acclimation. Valdramidis et al. (2006) used capillary tubes for applying the thermal stress. They reported a come-up time of 30 s using this technique. Hence, the heating rate was of ~60°C/min. Garre et al. (2018) observed for dynamic heating profiles with a heating rate higher than 15°C/min stress acclimation was irrelevant for *E. coli* CECT 515. Furthermore, van Zuijlen et al. (2010) compared microbial inactivation observed using capillary tubes and a Mastia thermoresistometer, obtaining similar results. We, thus, hypothesize that the heating rates applied by Valdramidis et al. (2006) are high enough to inactivate the cells before they are able to develop an acclimation, and that both technologies are comparable. Consequently, the parameters *D*(*T*_*ref*_), *T*_*ref*_ and *z* of the model by Garre et al. (2018) were fixed to the D and z-values estimated from isothermal experiments. The remaining model parameters (*a, c*, and *E*) were estimated using the data reported by Valdramidis et al. (2006) for a biphasic heating profile with a heating rate of 1.64°C/min, resulting in estimated values of 0.49 min^−1^ (0.02), 0.037°C (0.001) and 0.46 (0.01) for the parameters *a, E*, and *c*, respectively. The rest of the dynamic experiments reported by Valdramidis et al. (2006) were not involved in the fitting process and were used for model validation.

**Figure 2:**
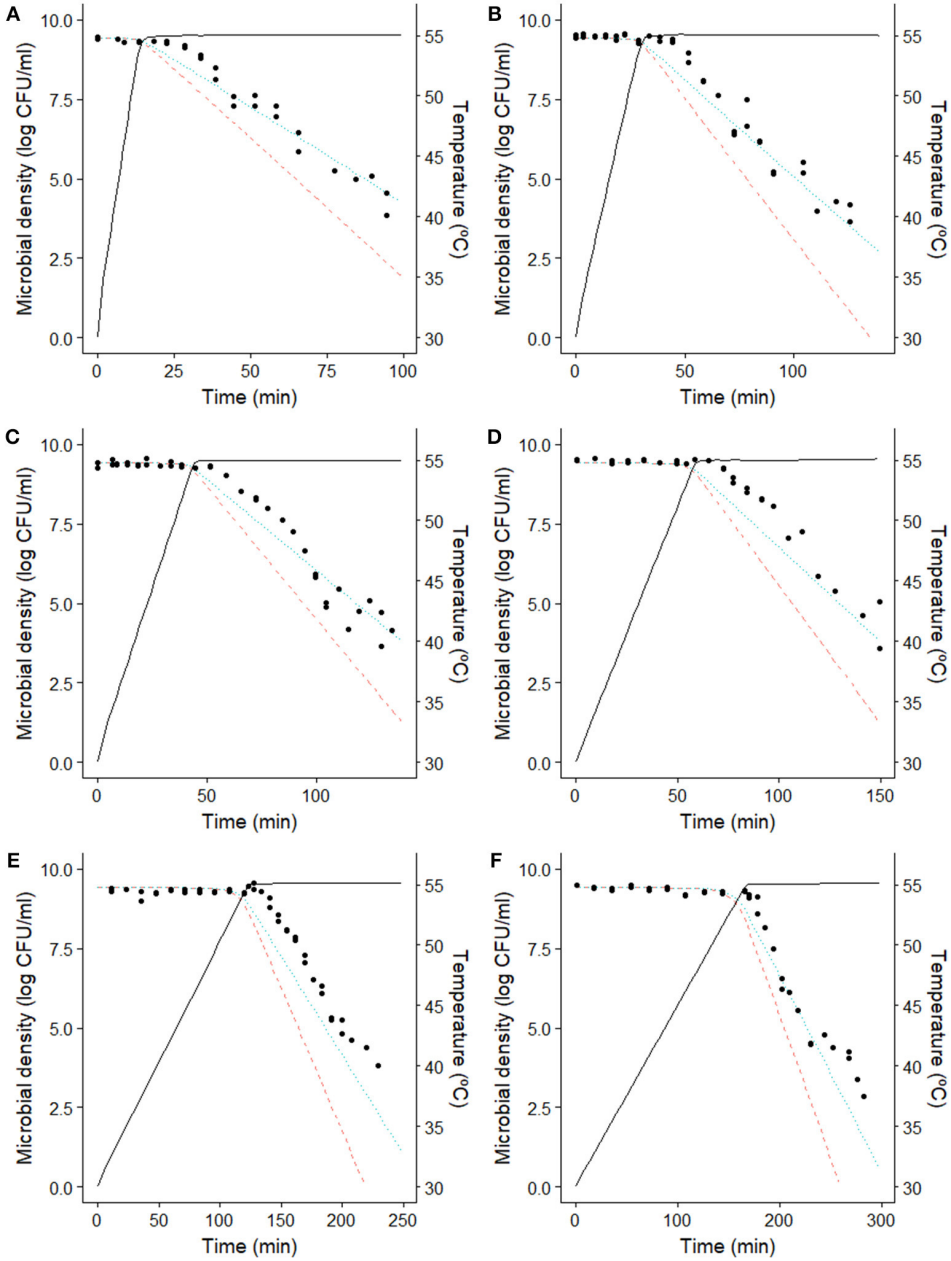
Comparison between the model predictions and the microbial counts of *E. coli* K12 MG1655 (Valdramidis et al., 2006) observed during the dynamic inactivation in BHI with a heating rate of **(A)** 1.64°C/min, **(B)** 0.82°C/min, **(C)** 0.55°C/min, **(D)** 0.40°C/min, **(E)** 0.20°C/min and **(F)** 0.15°C/min. (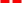) prediction of the Bigelow model using isothermal model parameters. (··) prediction of the model by Garre et al. (2018) fitted to the data reported by Valdramidis et al. (2006). (−) temperature profile.

Figure 2 compares the dynamic inactivation data reported by Valdramidis et al. (2006) for *E. coli* for six different dynamic profiles against the model predictions of the model by Garre et al. (2018) (dotted lines). The information provided by Figure 2 is complemented by Table 1, where the *ME, RMSE*, *A*_*f*_, and *B*_*f*_ for each prediction are reported.

**Table 1:** Indexes assessing the goodness of the model predictions for each model to the data of inactivation of *E. coli* K12 MG1655 on BHI reported by Valdramidis et al. (2006).

	***ME*** **(log CFU/ml)**		***RMSE*** **(log CFU/ml)**		***A***_*****f*****_		***B***_*****f*****_	

**Heating rate (**°**C/min)**	**Bigelow**	**Stress acclimation**	**Bigelow**	**Stress acclimation**	**Bigelow**	**Stress acclimation**	**Bigelow**	**Stress acclimation**
1.64	−1.00	−0.10	1.22	0.35	16.60	2.22	0.10	0.80
0.82	−1.12	−0.25	1.47	0.45	29.70	2.84	0.08	0.57
0.55	−1.01	−0.09	1.35	0.44	22.40	2.77	0.10	0.81
0.40	−0.97	−0.43	1.34	0.58	21.70	3.83	0.107	0.37
0.20	−1.54	−0.58	2.18	0.82	150	6.63	0.03	0.26
0.15	−1.48	−0.37	2.23	0.68	169.00	4.79	0.03	0.42

As already reported by Valdramidis et al. (2006), the *E. coli* strain used for the experiments was able to develop a stress resistance during the early, sub-lethal, stages of the treatment. Consequently, the Bigelow model overpredicts microbial inactivation for every dynamic temperature profile, with a mean error ranging between −0.97 and −1.54 log CFU/ml (Table 1). On the other hand, the model by Garre et al. (2018) fitted to the data by Valdramidis et al. (2006) is successful at describing the microbial response of every inactivation treatment (even those that were not used for model fitting). For the six temperature profiles tested, the model is able to predict the slope of the curve during the holding phase, with *ME* lower than 0.6 log CFU/ml and RMSE lower than 0.9 log CFU/ml for every profile (Table 1). Note, however, that the model overpredicts (fail-dangerous) the microbial inactivation at the beginning of the holding phase for every temperature profile tested (Figure 2). This is especially evident for the experimental data obtained at 0.20°C/min. This deviation is also observed in the study by Corradini and Peleg (2009), who analyzed the same data set. The reason for this might be the presence of a shoulder effect at the beginning of the holding phase which is not considered by the model used. Indeed, Valdramidis et al. (2006) observed a short shoulder for isothermal experiments, estimating a value of log *C*_*c*_(0) = 0.82 for the initial value of the variable describing the physiological state of bacterial cells in the Geeraerd model (Geeraerd et al., 2000). Despite this small deviation at the beginning of the holding phase, the model is successful at characterizing the overall microbial response taking into account the induced thermal resistance.

Variability of heat resistance among different microbial strains to the same treatment has been previously studied (den Besten et al., 2017). Traditionally, this comparison is performed by fitting a microbial inactivation model to experimental data and assessing statistical differences between the estimated model parameters. For instance, Hassani et al. (2006) performed non-isothermal inactivation experiments on different strains of *Staphylococcus aureus*, fitted the Mafart model (Mafart et al., 2002), and observed that the δ-value (time required to cause the first log-reduction) was dependent on the bacterial strain. This approach provides an overall understanding of the stress resistance of the bacterial population during the whole treatment. However, it does not allow to understand how the stress adaptation is developed, and how it affects the inactivation rate at each time point of the experiment. Several studies (Janssen et al., 2008; Dolan et al., 2013) have shown that the bacterial resistance during a thermal treatment does not depend only on the instantaneous temperature, but may be affected by the thermal profile. The development of stress acclimation (Hassani et al., 2006; Stasiewicz et al., 2008; Corradini and Peleg, 2009) is an example of such a situation. We believe that, in order to better understand the microbial response to the treatment, it is necessary to be able to discriminate between the thermal resistance due to the instantaneous environmental conditions (“static resistance”), and the effect of dynamic conditions, such as the development of a stress acclimation. The model proposed by Garre et al. (2018) specifies model parameters to describe the stress acclimation, providing further insight on its development and its impact on thermal resistance.

Table 2 compares the stress acclimation model parameters estimated here for strain K12 MG1655 against those reported by Garre et al. (2018) also using biphasic temperature profiles, but a different strain of *E. coli* (CECT 515) and PW media rather than BHI. Despite the fact that the temperature profiles used in both studies were different, the model by Garre et al. (2018) was in both cases able to describe the microbial inactivation experiments. Hence, the estimated model parameters are expected to be valid within the experimental range tested in both works. The model parameters estimated here for *E. coli* K12 MG1655 are significantly different to those reported for *E. coli* CECT 515. The value of *a* estimated for the K12 MG1655 strain is four times higher than the one estimated for the CECT 515 strain, whereas the value of *E* is a 6% lower. This implies that the transition between the two regimes shown in Figure 1 is faster for the CECT 515 strain. Once the temperature is high enough to enter the second phase, the rate of production of *p(t)* is higher for the K12 MG1655 strain. For *E. coli* K12 MG1655 in BHI a value of 0.47 (sd = 0.003) was estimated for *c*, whereas 1.98 (sd = 0.01) was estimated for *E. coli* CECT 515 in PW. Previous research works have shown that the microbial response to thermal treatments can vary between strains and/or heating media (Aryani et al., 2015; Aspridou and Koutsoumanis, 2015; Ros-Chumillas et al., 2017). Therefore, it is reasonable to consider that stress acclimation may also be dependent of these factors. Nevertheless, the aim of the present work was to describe differences in stress acclimation rather than to evaluate its impact depending on strain or heating medium. The impact of acclimation described exists and is only dependent on the experimental data evaluated, although it is reasonable to consider that some differences may be due not only to the strain, but also to the heating medium used for the experiments. Hence, although there is some uncertainty associated to the factors behind acclimation in both studies, they are out of the scope of this study and were not analyzed further.

**Table 2:** Comparison of the stress acclimation model parameter values and standard deviations estimated from the data of inactivation of *E. coli* K12 MG1655 on BHI reported by Valdramidis et al. (2006) against those estimated by Garre et al. (2018) for the inactivation of *E. coli* CECT 515.

	***E. coli* K12 MG1655**	***E. coli* CECT 515**
D-value (min)	5.67 (0.61) at 56.3°C	12.12 (0.52) at 52.5°C
z-value (°C)	4.11 (0.16)	5.12 (0.11)
*a* (min^−1^)	0.49 (0.002)	0.122 (0.002)
*E* (°C)	0.037 (0.001)	0.072 (0.001)
*c* (·)	0.46 (0.003)	1.98 (0.01)

**Figure 3:**
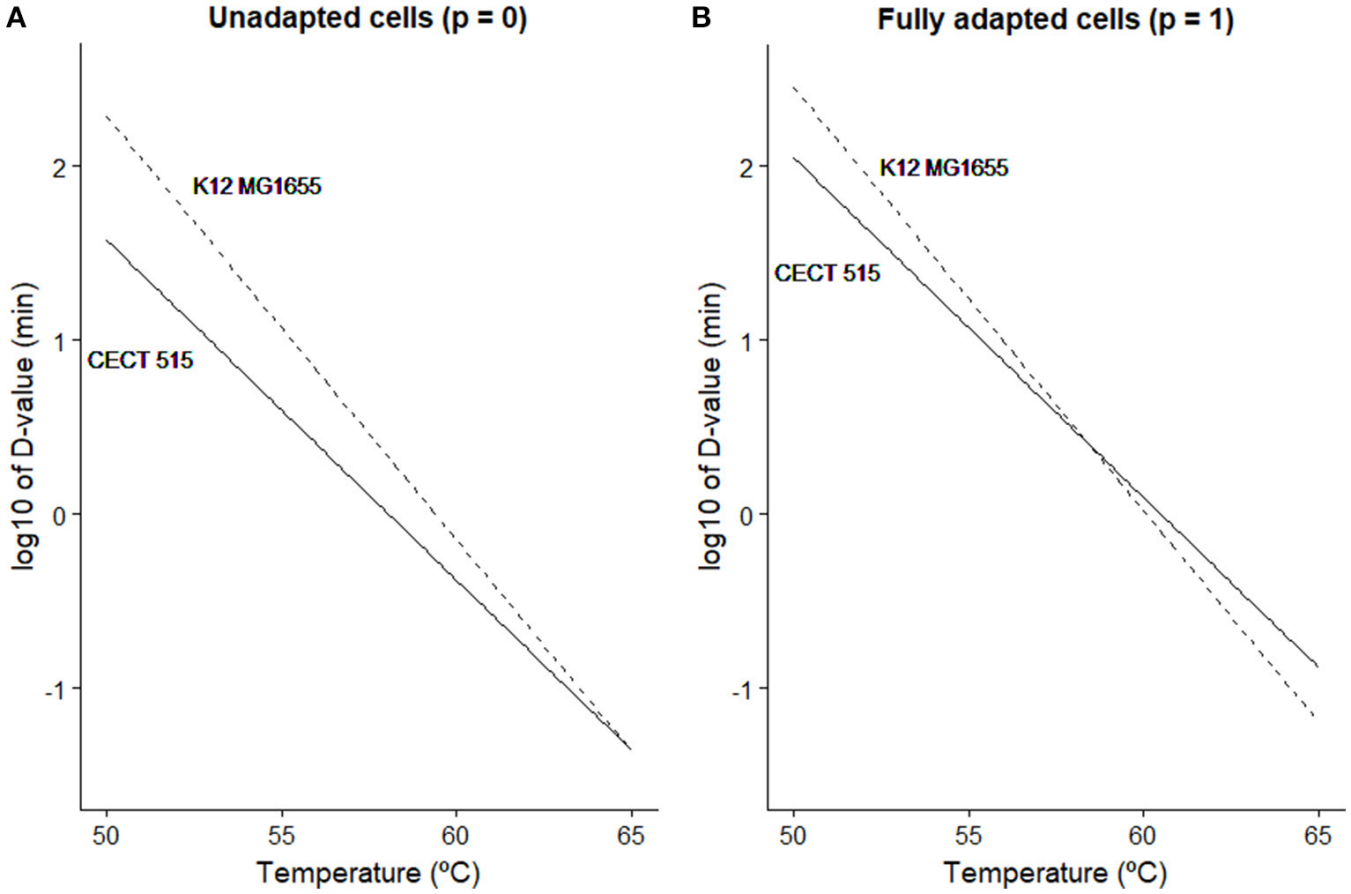
Comparison between the D-value predicted at different temperatures for *E. coli* K12 MG1655 (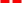) and *E. coli* CECT 515 (−) based on isothermal experiments **(A)** and taking into account the maximum stress resistance that the bacterial strain may develop **(B)**.

The model parameter *c* describes the maximum level of acclimation that the microbial population may develop. According to Equation (3), the D-value predicted by the Bigelow model is increased due to acclimation by a factor of (1 + *c*·(*t*)). Taking into account that the variable *p(t)* is bounded in the (0, 1) range, the maximum D-value is achieved when *p*=*1*. In that case, the D-value is increased by a factor of (1 + *c*). Hence, according to this model, *E. coli* CECT 515 may develop a stress acclimation increasing its stress resistance (estimated D value) almost a 300%, whereas for K12 MG1655, the increase in D-value is limited to 50%. In order to illustrate the impact that this effect may have for risk assessment, Figure 3 illustrates the D-values calculated in the 50-65°C range (typical inactivation temperature range for *E. coli*) for both strains taking into consideration or not the potential stress acclimation. In Figure 3A, D-values calculated only from isothermal experiments (static conditions) are illustrated. Based only on this information, strain K12 MG1655 would be considered more resistant to thermal treatment than CECT 515 for the whole temperature range and, thus, would be chosen as target for risk assessment calculations. Figure 3B has been constructed multiplying D-values (estimated using isothermal experiments) by the acclimation factor (1 + *c*), which represents the potential maximum increase in thermal resistance. At temperatures lower than 58°C, strain K12 MG1655 is more resistant to the thermal stress than CECT 515. However, for higher temperatures, the fact that CECT 515 strain has a higher *c*-value than K12 MG1655 results in the first strain being more resistant. Consequently, if processing conditions allow bacterial adaptation, CECT 515 strain may be more resistant to the thermal treatment than K12 MG1655, despite the greater resistance of the latter under static conditions.

**Figure 4:**
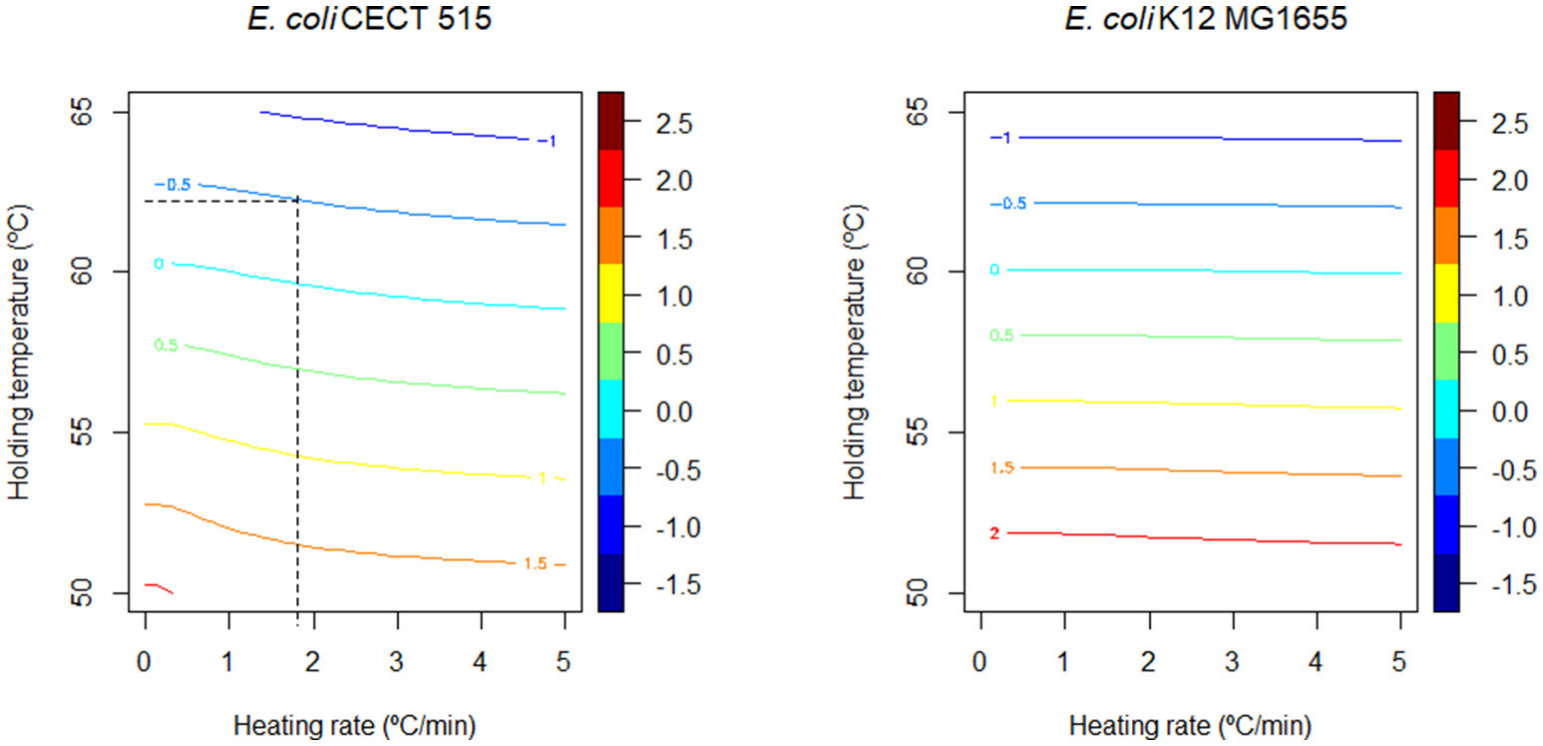
Comparison of the induced thermal resistance diagram obtained for *E. coli* CECT 515 in PW and *E. coli* K12 MG1655 in BHI. The black dashed lines represent the threshold heating rate at which strain CECT 515 becomes more resistant than the K12 MG16655 strain (see text).

Figure 4 shows the stress acclimation diagram constructed for both *E. coli* strains according to the methodology described in section Generation of Induced Thermal Resistance Diagram and the model parameters reported in Table 2. The curvature of the colored lines is higher for CECT 515 strain, indicating that the D-value of this strain at the end of the heating phase is strongly dependent on the heating rate because of its higher potential for stress acclimation (high *c*). By comparing the diagrams obtained for both strains, the strain with the higher D-value (taking into account stress acclimation) at the end of the heating phase can be identified for various combinations of *R* and *T*_*h*_. As already reported in Figure 3, for temperatures lower than 58°C, the D-value of the K12 MG1655 strain is higher than the one of CECT 515, regardless of the duration of the sub-lethal phase. However, for higher temperatures CECT 515 strain can be more resistant if the profile allows the development of stress acclimation. As shown by the dashed black line, at 62°C the D-value calculated for CECT 515 strain is higher than the one for K12 MG1655 strain if the heating rate is lower than 1.8°C/min. Higher heating rates do not allow the development of a stress acclimation, resulting in the strain K12 MG1655 being the one with the highest thermal resistance.

**Figure 5:**
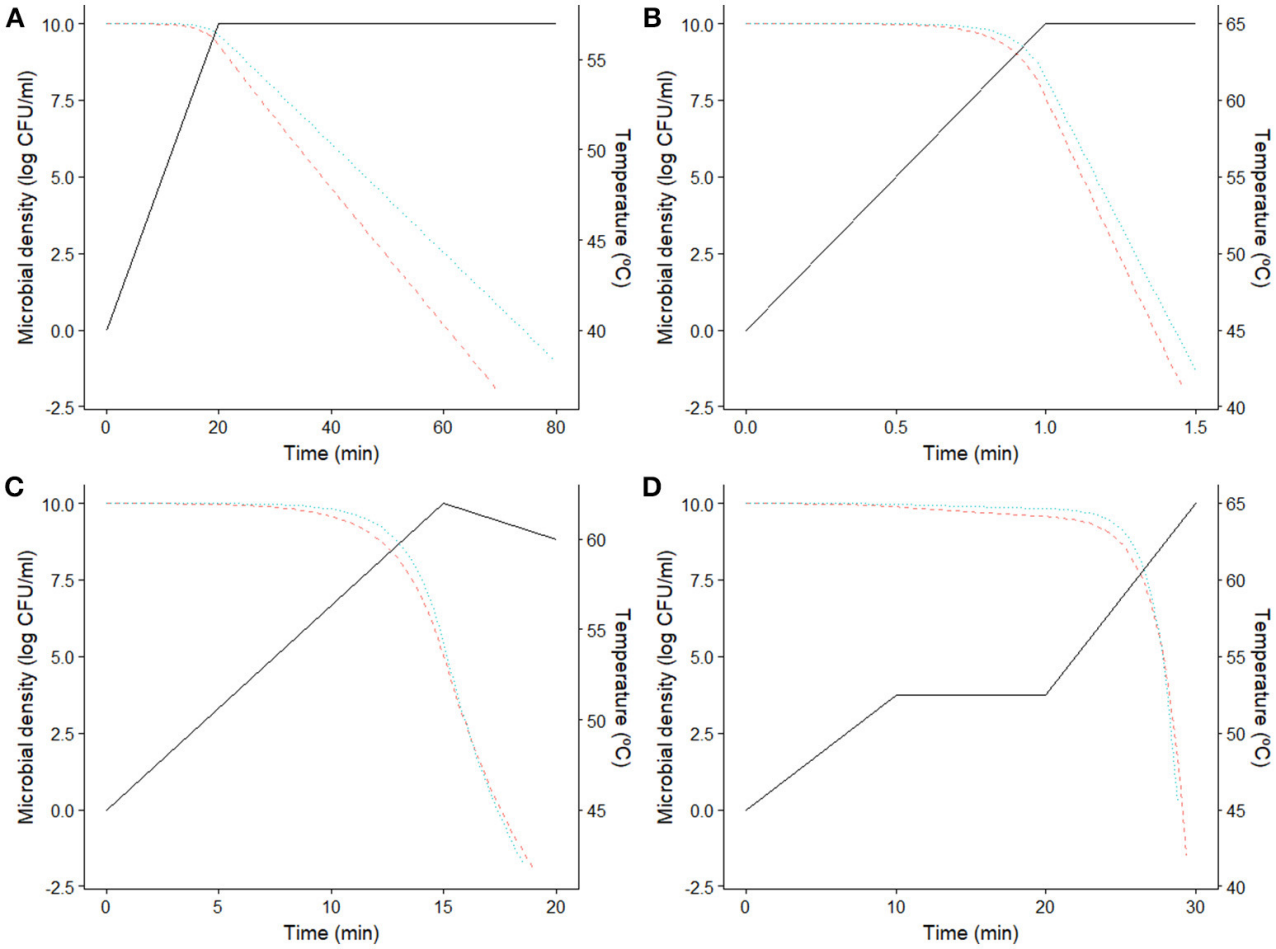
Predicted microbial density for *E. coli* CECT 515 (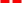) and K12 MG1655 (··) for four different dynamic thermal treatments (−). **(A)** Low temperature profile, **(B)** fast heating profile, **(C,D)** profiles with high temperature and slow heating.

The information provided by this diagram can help identifying the most resistant microorganism depending on processing conditions. However, the number of bacterial cells surviving the thermal treatment also depends on the prevalence data of the different strains, as well as the fraction of the population killed during the sub-lethal phase of the treatment. Hence, the results drawn from Figure 4 have to be complemented with numerical simulations or empirical data where the microbial response to the whole thermal treatment is studied. Figure 5 reports four different numerical simulations, showing the predicted microbial inactivation for both *E. coli* strains according to the models fitted assuming the same initial concentration for both. In Figure 5A, the temperature remains below 58°C for the whole treatment. Consequently, K12 MG1655 strain (blue, dotted line) would be the critical one (assuming the same prevalence for both strains), with higher D values than CECT 515 (red, dashed line) during the whole treatment. The profile simulated in Figure 5B reaches a holding temperature of 65°C (above the threshold temperature of 58°C). However, the heating phase of this treatment is very short, with a heating rate of 20°C/min, so bacterial cells are not able to develop a significant stress acclimation. Therefore, K12 MG1655 strain remains the most resistant one. This is not the case for the temperature profiles in Figure 5C,D, where the treatment reaches temperatures above the threshold temperature of 58°C and the heating phase is long enough for the bacterial cells to develop an adaptation. In consequence, during the first stages of the treatments, K12 MG1655 strain would have higher survival rates due to its higher static thermal resistance. However, at the end of the experiment strain CECT 515 has developed a higher stress acclimation and thus higher number of survivors at the end of the experiment. Hence, under these conditions this strain would be the most resistant one to these two treatments, despite having a lower resistance under isothermal conditions than strain K12 MG1655.

Therefore, the methodology followed in this work allows to evaluate whether stress acclimation can be relevant for a given thermal profile, and to identify the most resistant bacterial strain accordingly. This methodology can be used for risk assessment studies, where calculations are usually limited to the most resistant bacterial strain for the temperature profile studied. It can be summarized in the following steps:

1. Estimate D and z-values using isothermal experiments.
2. Estimate the model parameters of the model by Garre et al. (2018) describing stress acclimation (*a, E* and *c*) using non-isothermal experiments.
3. Plot the D-values calculated considering the stress acclimation and without considering it for the relevant temperature range (Figure 3).
  - If the D-value of bacterial strain “A” is higher than for strain “B” in the whole temperature range in both situations, strain “A” is more resistant to thermal treatments. In this case, no further investigation is required.
  - Otherwise, stress acclimation is relevant and further investigation is needed (continue with step 4).
4. Build the stress acclimation diagram for both bacterial strains. Compare them to identify combinations of temperature and heating rates where stress acclimation may be relevant.
5. Use numerical simulations or experimental data to validate the conclusions drawn in step 4.

## Conclusions

In this work, the relevance of stress adaptation for risk assessment has been evaluated. The microbial inactivation model of (Garre et al., 2018), which was validated on *E. coli* CECT 515, has been able to successfully describe the inactivation of *E. coli* K12 MG1655 according to the data published by Valdramidis et al. (2006). This model includes parameters and a variable to specifically describe the development of stress adaptation. Therefore, it has been used to compare the ability of both bacterial strains to develop a stress acclimation, concluding that it is strain dependent although other factors such as the heating medium can also have an impact. In spite of the K12 MG1655 strain having a higher resistance to thermal treatments where the heating is fast enough to inactivate the bacterial cells, the CECT 515 strain has a higher potential for stress acclimation. As a consequence, for treatment temperatures above 58°C, the CECT 515 strain may be more resistant if the thermal profiles allow for stress acclimation.

The mathematical calculations required to develop a quantitative microbial risk assessment are time demanding. For this reason, they are limited to a handful of bacterial strains considered the most resistant to the treatment (and/or better adapted for growth during storage). In every study published in the literature so far, this selection is always made taking into consideration only the static resistance of the bacteria according to isothermal treatments (D and z-values). The results obtained in this work demonstrate that stress acclimation can be relevant for some thermal profiles. Therefore, it must be considered when identifying the most resistant bacterial strain.

## Author contributions

PF and AG conceived the study. AP, AI, and JE coordinated the study. AG processed the data, implemented the methods with the help from JE and carried out all the computations. All the authors analyzed the results. AG, PF, and JE drafted the initial manuscript. All authors contributed to writing the final manuscript, reading, and approving the submitted version.

### Conflict of interest statement

The authors declare that the research was conducted in the absence of any commercial or financial relationships that could be construed as a potential conflict of interest.
